# Modelling of segmented high-performance thermoelectric generators with effects of thermal radiation, electrical and thermal contact resistances

**DOI:** 10.1038/srep24123

**Published:** 2016-04-07

**Authors:** Zhongliang Ouyang, Dawen Li

**Affiliations:** 1 Department of Electrical and Computer Engineering, Center for Materials for Information Technology, The University of Alabama, Tuscaloosa, AL 35487, United States

## Abstract

In this study, segmented thermoelectric generators (TEGs) have been simulated with various state-of-the-art TE materials spanning a wide temperature range, from 300 K up to 1000 K. The results reveal that by combining the current best p-type TE materials, BiSbTe, MgAgSb, K-doped PbTeS and SnSe with the strongest n-type TE materials, Cu-Doped BiTeSe, AgPbSbTe and SiGe to build segmented legs, TE modules could achieve efficiencies of up to 17.0% and 20.9% at Δ*T* = 500 K and Δ*T* = 700 K, respectively, and a high output power densities of over 2.1 Watt cm^−2^ at the temperature difference of 700 K. Moreover, we demonstrate that successful segmentation requires a smooth change of compatibility factor *s* from one end of the TEG leg to the other, even if *s* values of two ends differ by more than a factor of 2. The influence of the thermal radiation, electrical and thermal contact effects have also been studied. Although considered potentially detrimental to the TEG performance, these effects, if well-regulated, do not prevent segmentation of the current best TE materials from being a prospective way to construct high performance TEGs with greatly enhanced efficiency and output power density.

Thermoelectric generators (TEGs) convert heat directly into electricity by using the Seebeck effect^1^. TEGs are solid-state devices without moving parts, thus operating quietly, holding a long service life and requiring almost no maintenance^2^,^3^. Extensive studies have been conducted to make TEGs feasible in reality, however, thermoelectric (TE) technology is still far from being widely employed in practical applications^3^,^4^,^5^,^6^,^7^. One of the major issues that hinders TEGs from large scale production and popularization would be the low figure of merit (*ZT*) of thermoelectric materials^8^. Generally speaking, a *ZT* ~ 1 is needed for a TE material to be practical^9^. *ZT* is a temperature-dependent value and might vary drastically with little temperature change. Some prototype TE devices adopt TE materials with an average *ZT* below 1 over its operating temperature range, making the overall efficiencies of the devices far below 10%. TE materials have been widely studied and recent progress in *ZT* shows great promise in the development of new generation TEGs. Xie *et al*. developed p-type nanostructured (Bi,Sb)_2_Te_3_ bulk materials with an average *ZT* ~ 1.4 between 300 and 450 K^10^. Zhao *et al*. synthesized a p-type MgAgSb-based bulk material with a maximum *ZT* of ~1.4 at 475 K^11^. In 2014, Wu *et al*. created p-type K-doped PbTe_0.7_S_0.3_ bulk materials with a minimum and maximum *ZT* ~ 1.56 at 550 K and 2.2 at 800 K, respectively^12^. Also in 2014, Zhao *et al*. manufactured p-type SnSe single crystals with an impressive *ZT* of 2.6 ± 0.3 at 923 K, along with an average *ZT* well above 2 from 800 K to 975 K^13^. The development of n-type TE materials, on the other hand, has experienced a gentle progress instead of an equally rapid one as p-type TE materials^14^,^15^,^16^,^17^,^18^,^19^,^20^,^21^,^22^,^23^,^24^. In the low temperature range (300 K~500 K), Liu *et al*. fabricated Cu-Doped BiTeSe bulk materials maintaining an average *ZT* value slightly higher than unity^25^. In the mid-high temperature range, Hsu *et al*. exhibited a material system AgPb_m_SbTe_2+m_ possessing a maximum *ZT* of 2.2 at 800 K^26^. Beyond 800 K, Shi *et al*. and Basu *et al*. introduced multiple-filled skutterudites with *ZT* = 1.7 at 850 K^27^ and silicon germanium alloys (Si_80_Ge_20_) with *ZT* ~ 1.84 at 1073 K^28^, respectively. In addition to *ZT*, according to the theory, the ultimate efficiency of a TEG is determined and capped by the so-called Carnot efficiency *η*_*c*_ = (*T*_*h*_ − *T*_*c*_)/*T*_*h*_, where *T*_*h*_ and *T*_*c*_ are the temperatures of TEG’s hot side and cold side, respectively^29^. If *T*_*c*_ is kept at a constant temperature, for example, room temperature, then higher *T*_*h*_ will lead to higher ultimate efficiencies of TEGs. In other words, big temperature gradient across the TEG could yield a high-efficiency outcome, assuming that the employed TE material would not deteriorate drastically over a large temperature range.

Currently, no single TE material is qualified for this mission and different TE materials excel in their respective temperature ranges. One question arises naturally: is it possible to build segmented TEGs with various TE materials and make them cooperate with each other to result in an overall high performance? A few researchers have done some work in this respect, for example, Snyder *et al*. introduced a function called compatibility factor that characterizes the feasibility of combining two or more TE materials without having them adversely interacting with each other^30^,^31^. McEnaney *et al*. discussed the modelling of segmented TEGs using Bi_2_Te_3_ and Skutterudite^32^. Hadjistassou *et al*. described a design method of segmented Bi_2_Te_3_–PbTe TEGs in terms of comparing the average and collective Seebeck coefficient of Bi_2_Te_3_–PbTe to that of the pure Bi_2_Te_3_ and PbTe materials^33^. Ngan *et al*. provided an overview of theoretical efficiencies of segmented TEGs with various combinations of TE materials, by using a custom-made 1D numerical model^34^. However, there are few studies using the established 3D simulation environment, such as Ansys or Comsol, to accurately evaluate the performance of complex TEG modules with most up-to-date material combinations. For example, Xiao *et al*. analyzed one unicouple (a pair of p element and n element) model with bismuth telluride and filled-skutterudite^35^. Erturun *et al*. tested thermo-mechanical performance of four-leg models by using BiTe and CoSb-based Skutterudite^36^. One-unicouple models with various footprints were utilized by Rezania *et al*. to study the optimization of power generation based on p-type Zn_4_Sb_3_ and n-type Mg_2_Si_1−x_Sn_x_^37^. Nevertheless, these 3D models are simple in geometry with less number of TE unicoulples, and use out-of-date materials. Also their results were obtained without taking thermal transfer loss and contact effects of any kind into consideration.

Although segmenting approach is lucrative, it inevitably gives rise to some new issues. One is that segmentation introduces new interfaces between TE materials in addition to leg-electrode interfaces. These interfaces host electrical and thermal contact resistances, which not only incur net losses, for example, extra Joule heat, but can also cause temperature redistribution in the TEG leg, offsetting the optimal temperature range for each TE materials, thereby reducing the overall efficiency and output power. The other issue is the pronounced thermal transfer loss, which includes thermal convection and radiation loss. When aiming at higher efficiency with segmented structure, higher temperature has to be involved, leading to a possibly much greater level of thermal convection and radiation losses. The convection loss can be eliminated by appropriate insulation, thus it is not considered in this study^38^. The radiation and contact losses deserve serious attention for a successful construction of segmented TEGs. Most of the contact-related studies focused only on the interfaces between the TE materials and the electrodes^39^,^40^,^41^,^42^. Even with the segmented structure, just a single TEG leg with only one interface between two TE materials was investigated^43^. None of them considers contact resistances from both segment-segment and segment-electrode interfaces. Furthermore, to the best of our knowledge, there is no simulation study on the effect of the thermal radiation loss.

In this study, more sophisticated geometries with up to 128 unicouples (16 × 16 = 256 legs) are adopted to build symmetrical and non-symmetrical TEG models. Manifold selection of TE materials, covering both the moderate ones and the best ones, are employed to fulfill the simulation. The thermal and electric properties of the TE materials are all temperature dependent, spanning a wide temperature range, extracted directly from the recently published experimental data. Moreover, the segmentation compatibility has been confirmed for the combination of the current best p-type and the strongest n-type TE materials. On the basis of TEG model with the optimized p-n leg ratio, thermal radiation and contact resistances have been taken into account. Both the electrical and thermal contact resistances at segment-segment and segment-electrode interfaces are examined. In addition, thermal radiation effect has also been explored with the radiation level from zero to perfect blackbody. The results demonstrate that the TEG performance experiences plateaus at lower contact resistance ranges, indicating that if interfaces could be well controlled, the contact effects would not have remarkable influence on the TEG performance. Overall speaking, the segmentation of the best p-type TE materials and strongest n-type TE materials up to date provides a promising route to achieve a high performance TEG. All the simulations in this study are implemented by using the 3D finite element analysis (FEA) solver Ansys.

## Method

### Governing equations

To get the insight of numerical simulation, we have first derived the analytical solution for one dimensional TEG problem, involving n pairs of thermocouples and an external load with resistance *R*_*L*_. Each TEG thermocouple consists of one p element (leg) and one n element. In the derivation, all the thermal transfer loss, electrical and thermal contact resistances are ignored. When the system arrives at a steady state, the power absorbed at the hot side of the TEG module and the power released at the cold junction can be expressed as following^1^,^29^,


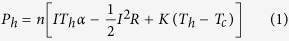



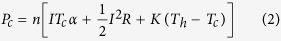


In both Equations (1) and (2), the first terms in the square brackets represent Peltier heat (power) generated, while the second and third terms denote Joule heat (power) and Fourier heat (power) transfer, respectively. The ratio1/2 in front of the Joule term indicates that each of the hot junction and cold junction “consumes” half of the total created Joule heat, since the TEG module has the same number of p-type elements as n-type elements. The Seebeck coefficient *α*, resistance of a thermocouple *R*, and thermal conductance *K* can be written more explicitly as


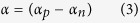



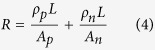



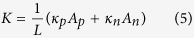


where the subscripts indicate p-type and n-type with L as the TEG leg length and A as the cross-section area of the TEG leg. As the difference between *P*_*h*_ and *P*_*c*_, the output power of the system can also be expressed in terms of the current and the external load resistance *R*_*L*_. In addition, the current in the system equals the Seebeck emf divided by the total resistance (internal *R* plus external *R*_*L*_).


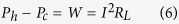



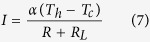


Combining Equations (1), (2), (6) and (7), the efficiency of the TEG module can be represented as,


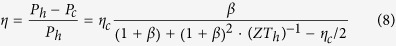


where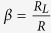, 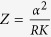
(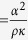 when referring to a single leg with sole TE material) and 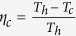. It can be shown that the maximum efficiency occurs at 
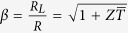
, where 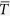 is the average of *T*_*h*_ and *T*_*c*_.


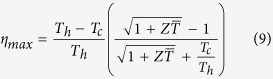


Equation (9) indicates that *η*_*max*_ increases monotonically with 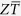. Once *T*_*h*_ and *T*_*c*_ have been chosen, the efficiency of the TEG module can be further optimized by maximizing *Z*. It is worth noting that all the above deductions are based on small temperature difference assumption: *T*_*h*_ − *T*_*c*_ = Δ*T* → 0. Under this prerequisite, the Seebeck coefficients, resistivities and thermal conductivities of both n and p-type semiconductors can be deemed as constants. As a result, it is not hard to verify that *Z* would reach its extreme value when the following relation is established.


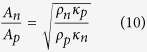


Generally speaking, a single TE material could be depicted by the dimensionless figure of merit *ZT*, defined as *ZT* = *α*^2^*T*/*ρκ*, and the optimal efficiency is still determined by Equation (9).

The 1D mathematical model presented above can only be used to get analytical solution under small temperature difference assumption: *T*_*h*_ − *T*_*c*_ = Δ*T* → 0, or in other words, constant thermoelectric properties of the TE materials. However, when temperature-dependent TE properties are involved, only numerical solution could be obtained. In this study, the coupled thermoelectric equations used by the FEA solver are


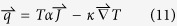



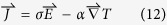


where 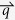 is the heat flux, 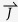 is the electric current density, *α* is the Seebeck coefficient, *σ* is the electrical conductivity, *κ* is the thermal conductivity, 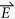 is the electric field and *T* is the absolute temperature.

### Material properties

**Figure 1:**
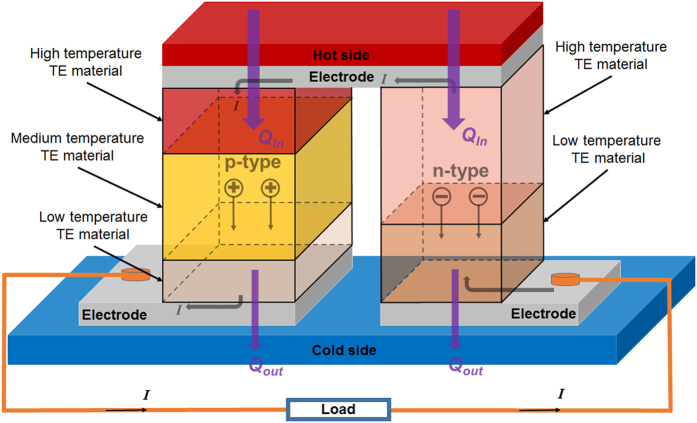
A schematic TEG model with segmented legs.

From a wide selection of TE materials, we choose the best p-type TE materials so far, covering different temperature ranges, from Bi_2_Te_3_ alloy for room temperature, to MgAgSb for mid-temperature, and to PbTeS and SnSe for high-temperature application. On the other hand, three distinctive n-type material combinations are used for comparison and conclusion without loss of generality. Table 1 shows a detailed temperature dependence of *ZT*s and Seebeck coefficients of those TE materials used in this study. It is worth mentioning that, however, the thermoelectric properties that are input directly into the simulation, are Seebeck coefficients, electrical resistivities and thermal conductivities, which are all temperature dependent and listed in Supplementary Table S1. Under the p-type and n-type 1 categories are the TE materials with the best performance to date. Figure 1 illustrates the segmented TEG unicouple with three p-type materials and two n-type materials, and the TEG legs are connected thermally in parallel but electrically in series. In this study, more or less TE materials are chosen for both p-type and n-type legs based on the temperature difference between the hot side and the cold side.

In addition, copper is used for electrodes. Its thermoelectric properties are also temperature dependent as shown in supporting material (Supplementary Table S2). Through all the simulations, the cold side temperature of the TEG models is set to 300 K (room temperature), while the hot side temperature is chosen to be 500 K, 800 K and 1000 K.

### TEG leg geometries

The performance of TEGs with various leg geometries has been investigated to study the possibility of segmenting different TE materials to form a high-efficiency TEG device. The TEG models are divided into two categories: symmetrical models, i.e. p-type and n-type legs sharing the same geometry, and non-symmetrical models, in which p-leg and n-leg have different cross-sectional area as shown in Supplementary Figure S1. All the symmetrical models are built with an overall active cross-section area of 1474.56 mm^2^ (the summation of the cross-section area of all the TEG legs). Total leg numbers varies from 16 (4 × 4), 64 (8 × 8), 144 (12 × 12) to 256 (16 × 16), which correspond to single leg dimensions (either p- or n-type) of 9.6 mm × 9.6 mm, 4.8 mm × 4.8 mm, 3.2 mm × 3.2 mm and 2.4 mm × 2.4 mm, respectively. Four different leg thicknesses are utilized, including 3 mm, 10 mm, 15 mm and 20 mm.

In modelling non-symmetrical leg geometries, the cross-sectional area of one type of legs is fixed, while the dimensions of the other type of legs change. Therefore this category can be further classified into two groups: group 1 with fixed p-type leg dimension of 9.6 mm × 9.6 mm, while n-type legs adopt cross-sections from 2.4 mm × 2.4 mm to 8.4 mm × 8.4 mm; group 2 with n-type legs set at 9.6 mm × 9.6 mm and p-type legs ranging from 2.4 mm × 2.4 mm to 8.4 mm × 8.4 mm. These non-symmetrical models have 2 × 2 legs and share the same leg length of 10 mm. The non-symmetrical geometries used in the simulation are listed in Supplementary Table S3. The symmetrical dimensions with *A*_*p*_ = *A*_*n*_ = 9.6 mm × 9.6 mm are also included in the table for comparison.

## Results and Discussion

### Symmetrical models

In modelling symmetrical TEG modules, the state-of-the-art p-type materials BiSbTe, MgAgSb, PbTeS and SnSe are combined for p-type legs, and identical resistivities, thermal conductivities, and Seebeck coefficients flipped to negative values are used for n-leg modelling. For a temperature difference of Δ*T* = 200 K (300 K ~ 500 K), TEG legs are segmented with BiSbTe and MgAgSb. In such temperature gradient, the two material segments are enough to ensure a uniformly high *ZT* across the legs. Since no single TE material is able to keep a *ZT* greater than unity over a large temperature range, as the Δ*T* increases, more TE materials are required to advance thermoelectric energy conversion over large temperature difference. Thus the third and fourth layers of TE materials are added to TEGs for Δ*T* = 500 K and 700 K.

**Figure 2:**
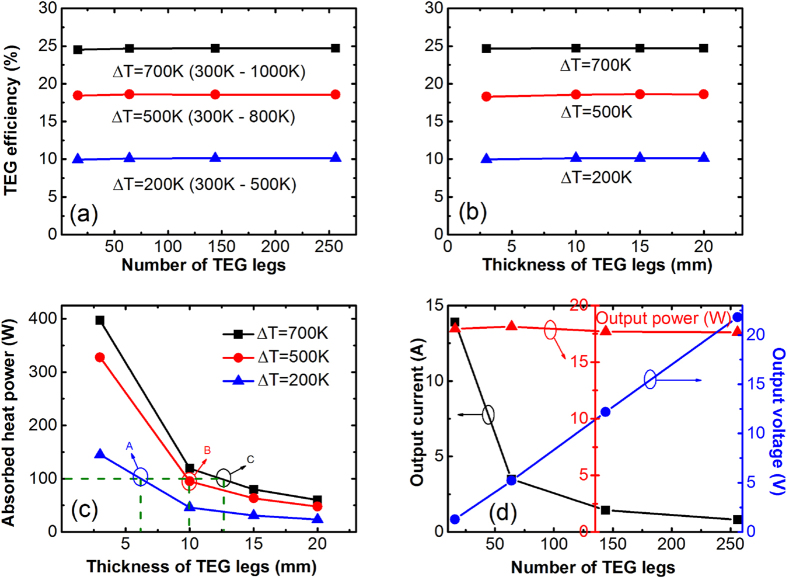
(**a**) Efficiency of thermoelectric modules versus total number of TEG legs. The leg thickness is set to be 10 mm. (**b**) Efficiency versus thickness of TEG legs, which is based on 256 (16 × 16) total number of TEG legs. (**c**) The required heat power versus leg thickness with various temperature differences. (**d**) Effects of number of TEG legs on output current, output voltage and output power. The temperature difference Δ*T* = 500 *K*, and leg thickness L = 10 mm.

Figure 2a,b show simulation results of thermoelectric energy-conversion efficiency as the total number of TEG legs and leg thickness vary. This efficiency from symmetrical modelling is actually the so-called leg efficiency since only the properties of p-type materials are used. Neither the TEG leg thickness nor the total number of TEG legs has any significant influence on the efficiency of the TE modules. This conclusion verifies the 1-D analytical result in Equation (9) that there is no explicit term related to geometric factors, such as the total number of TEG legs and leg thickness. With adopted segmentation of TE materials, the leg efficiency of the TE modules depends heavily on temperature difference between the hot side and the cold end. For Δ*T* = 200 K, 500 K and 700 K, the TEG leg efficiencies are around 10.0%, 18.6% and 24.7%, respectively. These results approximate the theoretical upper limits, indicating that these materials are compatible as the segmentation follows a certain sequence^30^,^31^,^34^.

In addition to the efficiency, the heat absorption and output power were also studied for the symmetrical models. Figure 2c shows heat absorption rates at the TEG hot side as a function of leg thickness for various temperature differences. For any given temperature difference, as the TEG leg thickness increases, the required heat absorption decreases. The underlying principle is straightforward: a longer TEG leg is more difficult for heat to pass through, and thus easier for the TEG hot side to accumulate heat. As a result, less input heat power is required to reach or maintain the same temperature difference. From another perspective, if the input heat flux is constant, for instance, the exhaust heat from an engine, a longer leg length will create a greater temperature difference, thus leading to higher efficiencies. As demonstrated in Fig. 2c, for TEGs with leg thickness of ~6 mm, 10 mm and 13 mm, an input heat power of 100 W can produce a temperature gradient of 200 K (point A), 500 K (B) and 700 K (C), respectively.

Figure 2d shows that the output power barely experiences any change as the total number of TEG legs varies. As the total number of TEG legs increases, more pairs of p and n legs are connected in series, resulting in enhanced output voltage but reduced output current, keeping the output power almost the same. In practice, the number of TEG legs will mainly be determined by the load resistance, since the maximum efficiency and the maximal output power occur at 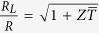 and 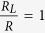, respectively. In other words, a larger load resistance requires a greater internal resistance of the TEG. For a given available surface area of a heat source, a high-performance TEG module can be achieved by shrinking down the cross-section area of individual leg thereby building more TEG legs.

### Non-symmetrical models

**Figure 3:**
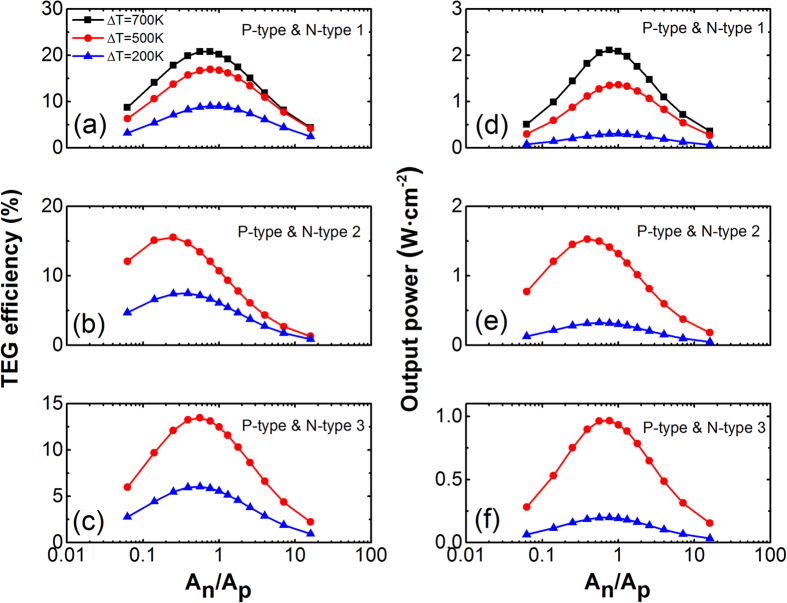
TEG efficiency (**a–c**) and output power per unit area (**d–f**) at different geometrical ratios. For all three TEG modules, the maximum efficiency is indeed achieved using non-symmetrical cross-section areas. The non-symmetrical TEG modules are built with the best p-type TE materials along with (**a,d**) n-type 1, (**b,e**) n-type 2, and (**c,f**) n-type 3 TE materials.

Non-symmetrical TEGs are modelled using the same p-type materials with three different combinations of n-type materials as listed in Table 1. Three temperature differences, Δ*T* = 200 K, Δ*T* = 500 K and Δ*T* = 700 K, are used in the simulation. The combination of the best p-type TE materials with the strongest n-type TE materials could yield an efficiencies of up to 17.0% and 20.9% at Δ*T* = 500 K and Δ*T* = 700 K, respectively. The simulation results also show that the maximum efficiencies are achieved by non-symmetrical TEGs for all three combinations (shown in Fig. 3a–c), given that p-type and n-type materials are not the same. Since the n-type materials are universally weaker compared to their p-type counterparts, the peak performance of the TEG modules emerges when the p-type legs have larger cross-sectional area than the n-type legs. Similar to efficiency, the relationship *A*_*n*_ < *A*_*p*_ is also necessary for the maximum output power per unit area, which is in good agreement with a previous study^37^. In addition, the output power densities can reach and exceed 2.1 Watt cm^−2^ at optimal geometrical ratio with a temperature difference of 700 K, as shown in Fig. 3d. Even with Δ*T* = 500 *K*, the output power densities can far surpass 1.0 Watt cm^−2^. This capability of generating high power density will have great impact on utilizing vehicles’ exhaust heat. For example, assuming that an exhaust pipe with 1m length and 10 cm diameter is completely covered with the proposed TEG device, the output power can be more than 6 kW, given that the exhaust temperature is around 500 °C, As noticed from Fig. 3, the maximum efficiency and peak output power do not concur at the same optimal geometrical ratio, since their triggering conditions are different: 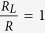 for the maximum output power, and 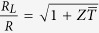 for the peak efficiency. In addition, since less cross-section area of n-type legs is required for reaching peak performance, non-symmetrical models would need less TE materials as compared to the symmetrical model, especially when the leg thickness is large, therefore leading to an economical design.

Nevertheless, above results indicate that the maximum efficiency and output power density are indeed achieved by non-symmetrical TEGs, given that p-type and n-type materials are not the same. This conclusion is supported by Equation (10), although it only applies when temperature difference approaches zero (Δ*T* → 0 To find the optimal cross-section ratio *A*_*n*_/*A*_*p*_ at which the maximum efficiency could be achieved in a segmented leg structure, the Equation (10) that associates the extensive quantity (cross-section area *A*_*n*_ and *A*_*p*_) with the intensive properties of materials (electrical resistivity *ρ* and thermal conductivity *κ*) has to be modified,


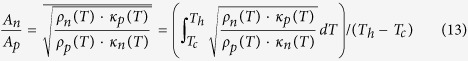


where 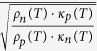 is the average over temperature range from *T*_*c*_ to *T*_*h*_. If *ρ*(*T*) and *κ*(*T*) of the TE materials are known in the operating temperature range, Equation (13) can be used to accurately calculate the optimal TEG leg geometries. However, in reality *ρ*(*T*) and *κ*(*T*) are typically discrete values obtained from the experiment. As a consequence, the integrations in Equation (13) should be replaced by summation, such as


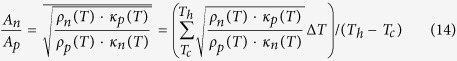


In this work, a temperature interval of Δ*T* = 50 *K* is adopted, alike most of the TE-related publications. Table 2 compares the optimal geometrical ratios *A*_*n*_/*A*_*p*_ between 3D simulation and estimation from Equation (14). The ratios from calculation and simulation match well with deviation less than 10%, which confirms the feasibility of the proposed Equation (14) for TEGs with segmented legs. To reach the highest TEG efficiency with given material combination, Equation (14) can be utilized to estimate the optimal geometrical ratio of TEG legs before conducting the simulation for guiding experiment.

### Compatibility of segmented TE materials

**Figure 4:**
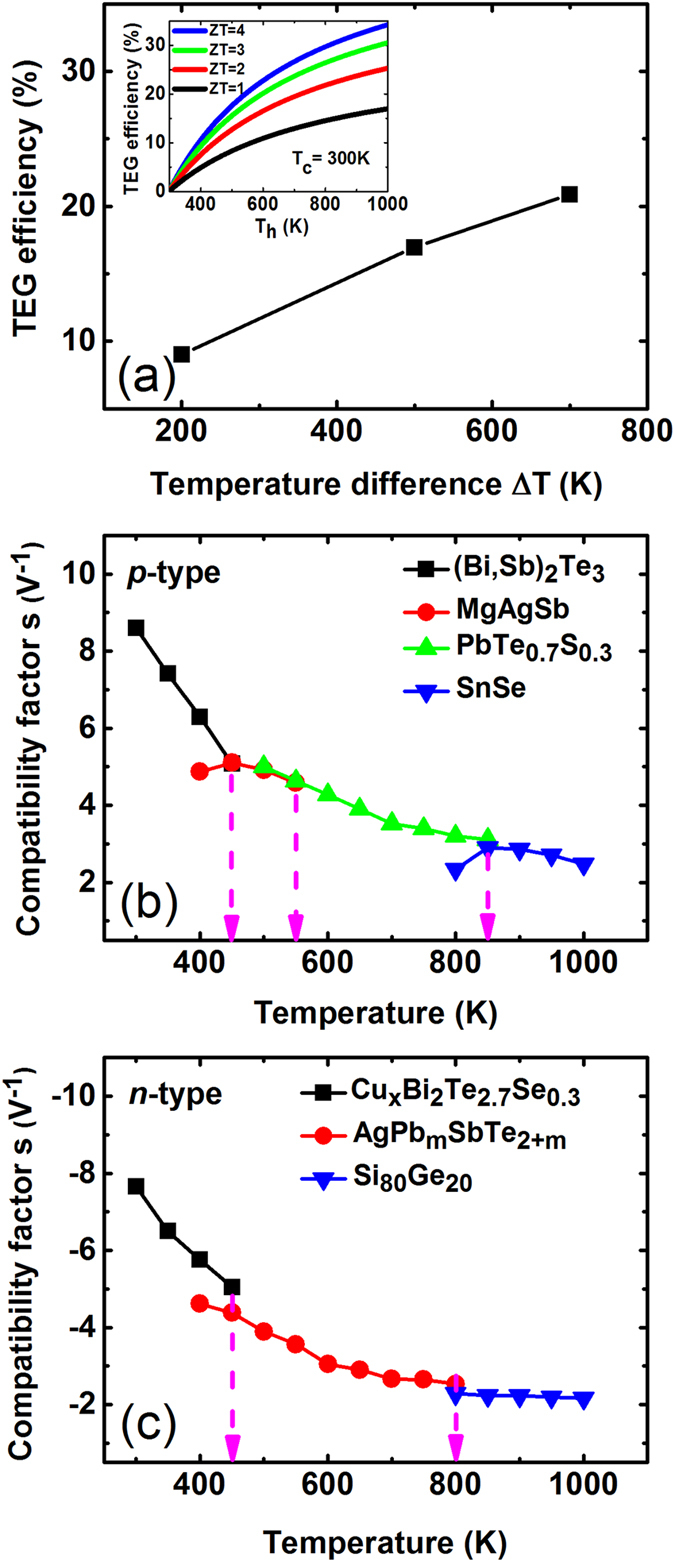
(**a**) TEG Efficiency vs. temperature differences for TEGs with the combination of the current best p-type and the present strongest n-type TE materials (inset: TEG efficiency vs. hot side temperature at different *ZT* values, where cold side temperature has been set to 300 K). (**b,c**) Compatibility factors of the series of best p-type and strongest n-type TE materials, respectively. The dashed lines indicate the interfaces between segments.

From the simulation, the combination of the current best p-type and the present strongest n-type TE materials can yield efficiencies of up to 9.0%, 17.0% and 20.9% respectively with temperature differences of 200 K, 500 K and 700 K, as shown in Fig. 4a. These efficiencies are close to the theoretical upper limits of TE materials with *ZT* = 2 as exhibited in the inset of Fig. 4a (deduced from Equation (9)), indicating that these TE materials are compatible and suitable to form segmented TE legs. According to the definition of the compatibility factor from Snyder *et al*., 
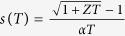
, any two TE materials with *s* values differing by a factor of 2 or more could not be connected to yield an effective segmentation^30^. Notice that *s* is temperature dependent and thus not unique for any TE material in its working temperature range. Based on *ZT* values and Seebeck coefficients in Table 1, the compatibility factors of the best p-type and n-type TE materials used in the simulation have been estimated, as exhibited in Fig. 4b,c, respectively. Although p-type SnSe doesn’t match directly with (Bi,Sb)_2_Te_3_/MgAgSb, since their compatibility factors differed by more than a factor of 2, however, the intermediate PbTe_0.7_S_0.3_ segment has appropriate *s* values that are close to those of SnSe and (Bi,Sb)_2_Te_3_/MgAgSb at the overlapping temperatures. Besides, PbTe_0.7_S_0.3_ is self-compatible^30^ in its own operating temperature range, i.e., its *s* value experiences only a mild variation (less than a factor of 2) in this temperature range. Therefore, the compatibility factor of the entire p-leg transformed smoothly from 300 K to 1000 K. The similar smooth transition of *s* value can also be observed for n-type TE materials in Fig. 4c. This suave evolution of compatibility factor guarantees a successful segmentation even if the *s* values at the two ends (cold side and hot side) differ by more than a factor of 2.

Figure of merit *ZT* is typically the first property to look at when choosing TE materials for segmentation purpose. The second most important parameter for segmentation, the compatibility factor, should also be given enough attention. As demonstrated in the Supplementary Figure S2, only high *ZT* will not ensure high efficiency. Figure S2a shows that the addition of a layer BaLaYbCo_4_Sb_12_, which has the highest *ZT* value of 1.66 around 850 K, reduces the optimal TEG efficiency instead of increasing it. Figure S2b clearly illustrated that *s* values of BaLaYbCo_4_Sb_12_ do not match well with compatibility factors of other segmented materials. This result verifies that compatibility factor cannot be ignored when segmenting different TE materials. In summary, when taking a TE material for segmentation, not only *ZT* value but also compatibility factor have to be examined. A smooth change of *s* value from one end of the TEG leg to the other is needed for a successful segmentation of different high-*ZT* TE materials, even if the change in *s* is more than a factor of 2 from the cold side to the hot side. Besides, each segmented TE material should have its own *s* value varying less than a factor of 2 in its operating temperature range.

### Thickness of the individual segments

**Figure 5:**
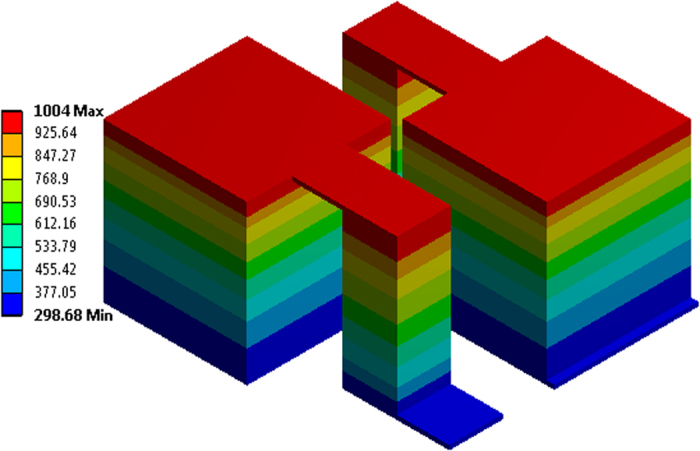
Temperature distribution in a 3D non-symmetrical TEG module, *A*_*n*_/*A*_*p*_ = 0.141, with the best p-type TE materials and 3 layers of the strongest n-type TE materials.

The simulation results can also be used to guide the fabrication of the TEG devices. Figure 5 shows the temperature distribution in a non-symmetrical TEG module with cold side at 300 K and hot side at 1000 K, from which the thickness of different TE materials in the leg can be determined based on their optimal temperature ranges. If the TEG leg length changes and the temperatures of both the hot side and cold end remain the same, the thickness of each individual layer can still be estimated based on the relative thickness percentages of the respective TE materials from the numerical simulation while maintaining the TEG efficiency.

For the TEG model with the optimized leg geometry, i.e. 9.6 mm × 9.6 mm for p-legs and 7.8 mm × 7.8 mm for n-legs, of the best TE materials’ combination, thermal radiation, electrical and thermal contact resistances have been added as boundary conditions. The four segmented p-type TE materials and three n-type TE materials introduce 9 interfaces per unicouple, including 5 segment-segment and 4 segment-electrode interfaces.

### Thermal radiation loss

**Figure 6:**
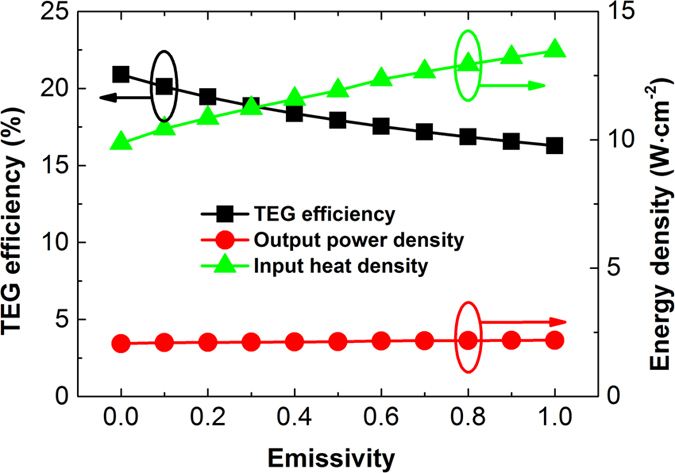
TEG efficiency, output power density and input heat density vs. emissivity/thermal radiation.

In modelling thermal radiation loss, emissivity from 0 to 1 are adopted, which corresponds to zero radiation loss and total black body radiation, respectively. Figure 6 shows that TEG efficiency decreases monotonically with the increase of the emissivity. With the highest level of radiation loss, the TEG efficiency falls to around 16.3%, which is still much higher than the efficiency of the currently state-of-the-art TEGs. The output power density stays nearly the same, which is due to the gain of the input heat power density for keeping the preset end temperatures of the TEG module. Although not exhibited here, the thermal radiation of any level does not have a noticeable effect on the temperature distribution of the TEG module, therefore it is not necessary to optimize the thicknesses of individual segmentations through iteration to obtain the best performance. It is worth mentioning that in our simulation the radiation is net loss without considering the reabsorption by the adjacent TEG leg faces. With reabsorption, the TEG efficiency could be slightly higher than that shown in Fig. 6 but will not exceed the point at zero emissivity. According to Stefan-Boltzmann Law and Second Law of Thermodynamics, a good emitter is also a good absorber and vice versa. If the emissivity is low, then the reabsorption will be weak and have little influence on the TEG performance. In the contrary, at high emissivity, the reabsorption will be strong, but it could never overturn the massive loss induced by the high level of radiation. Therefore, here we only consider thermal radiation as net loss to obtain the ultimate values.

### Contact resistances

**Figure 7:**
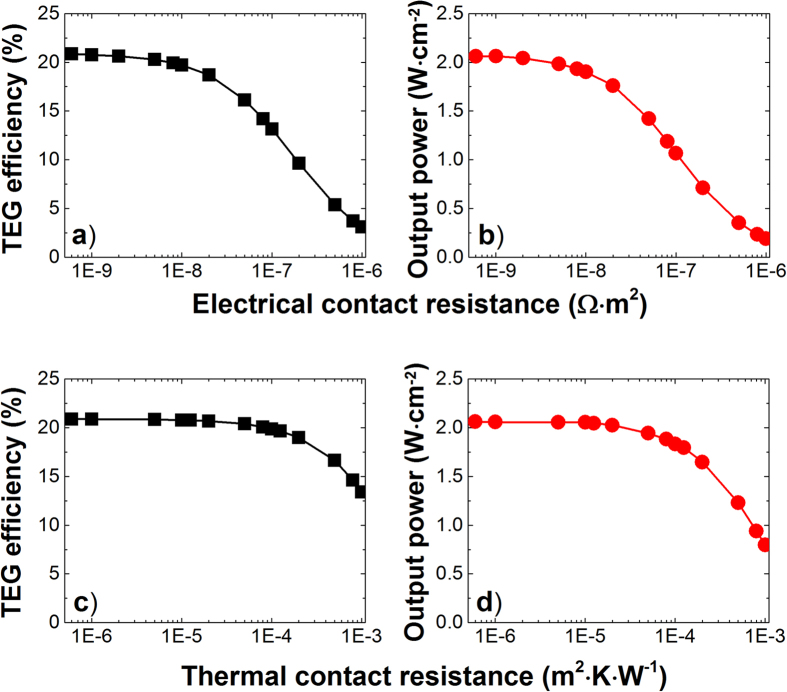
(**a**) TEG efficiency and (**b**) output power density vs. electrical contact resistance. (**c**) TEG efficiency and (**d**) output power density vs. thermal contact resistance.

In this study, electrical and thermal contact resistances at both segment-segment and leg-copper interfaces have been considered. The electrical contact resistance has been reported to have typical values falling in the range of 1 × 10^−9^ − 1 × 10^−7^ Ω · *m*^2 ^46. Figure 7a,b shows the influence of such electrical contact resistance on the TEG efficiency and output power density. As anticipated, when the electrical contact resistance increases, both efficiency and out power decreases, although there exists a plateau for electrical contact resistance less than 1 × 10^−8^ Ω · *m*^2^. With growing of the electrical contact resistance, the temperature distribution profile of the TEG legs is found to change accordingly. The thicknesses of each individual segmentation have been optimized iteratively to ensure that the physical interfaces match with the corresponding temperature distribution.

On the other hand, thermal contact resistance at interfaces varying in the range of 1 × 10^−6^ − 5 × 10^−4^ *m*^2^ · *K* · *W*^−1^ have been reported^47^. Figure 7c,d shows the thermal contact resistance does not have significant effect on the efficiency and output power density at the values less than 5 × 10^−4^ *m*^2^ · *K* · *W*^−1^, only beyond which the performance of the TEG module degrades rapidly. Similar to the influence from electrical contact resistance, as the thermal contact resistance increases, the temperature distribution profile also changes accordingly. The thicknesses have to be optimized iteratively to achieve the temperature distribution coincidental with the physical interfaces.

## Conclusion

A series of 3D TEG modules, including symmetrical and non-symmetrical models with diversified geometries and up to 128 unicouples, have been established in Ansys environment. Manifold TE materials, whose signature properties, such as the Seebeck coefficients, electrical resistivities and thermal conductivities are all temperature dependent and extracted directly from recent publications. It is found that the TEG modules with the current best p-type TE materials teamed up with the strongest n-type TE materials could yield efficiencies of up to 17.0% and 20.9% at Δ*T* = 500 K and Δ*T* = 700 K, respectively. The achieved high efficiencies approximate the theoretical efficiency upper limits, validating that the best p-type SnSe and the strongest n-type SiGe in the high temperature realm, hold the potential to combine with the traditional high-*ZT* low-temperature materials, such as p-type BiSbTe and n-type CuBiTeSe. Bridged by the intermediate segments for the sake of the compatibility, such combination is able to create high-performance TEG devices without adversely affecting each other between components. In addition, the output power densities over 2.1 Watt cm^−2^ are feasible at optimal geometrical ratio with a temperature difference of 700 K, even with Δ*T* = 500 *K*, the output power densities can reach and exceed 1.0 Watt cm^−2^. Due to the fact that the n-type TE materials are universally weaker than their p-type counterparts, unsymmetrical geometry of *A*_*n*_ < *A*_*p*_ is necessary in obtaining the optimized TEG performance. Results also show that the proposed relationship 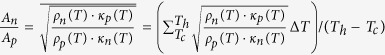 can be used to accurately speculate the optimal geometrical ratio for the maximum efficiency of TEG modules. From the perspective of the compatibility factor, a successful segmentation of different TE materials can be achieved by a gradual change of *s* value from one end of the TEG leg to the other, even if *s* of the cold side and the hot side differ by more than a factor of 2. In addition, the influence of thermal radiation and contact resistances has also been investigated. The results show that thermal radiation has limited effect on the TEG performance while contact resistances, particularly the electrical one, could have destructive impact on the TEG efficiency and output power. Nevertheless, the plateaus showing at lower contact resistances provide tolerance space for interface quality in building high performance segmented TEGs from the current best TE materials.

## Additional Information

**How to cite this article**: Ouyang, Z. and Li, D. Modelling of segmented high-performance thermoelectric generators with effects of thermal radiation, electrical and thermal contact resistances. *Sci. Rep*. **6**, 24123; doi: 10.1038/srep24123 (2016).

## Supplementary Material

Supplementary Information

## Figures and Tables

**Table 1:** Temperature dependence of *ZT*s and Seebeck coefficients for used TE materials.

	p-type		n-type 1		n-type 2		n-type 3	
	300–400 K BiSbTe^10^ 450–500 K MgAgSb^11^ 550–800 K KPbTeS^12^ 850–1000 K SnSe^13^		300–400 K CuBiTeSe^25^ 450–800 K AgPbSbTe^26^ 850–1000 K SiGe^28^		YbCoSb^44^		Pb(S,Se,Te)^45^	
*T* (K)	*ZT*	*α*(*μV*)	*ZT*	*α*(*μV*)	*ZT*	*α*(*μV*)	*ZT*	*α*(*μV*)
300	1.38	210	1.04	−186	0.38	−122	0.07	−50
350	1.47	220	1.06	−192	0.45	−128	0.12	−60
400	1.49	230	1.09	−194	0.55	−138	0.20	−75
450	1.36	233	1.06	−220	0.65	−143	0.29	−90
500	1.38	220	1.18	−245	0.75	−151	0.40	−108
550	1.56	235	1.34	−270	0.88	−158	0.53	−123
600	1.80	263	1.43	−305	1.00	−166	0.66	−140
650	1.95	283	1.64	−330	1.09	−173	0.79	−158
700	2.05	303	1.73	−350	1.18	−179	0.90	−175
750	2.18	308	1.94	−360	1.25	−183	0.95	−192
800	2.20	308	2.10	−375	1.33	−187	1.01	−205
850	2.39	340	1.40	−288	N/A			
900	2.53	340	1.50	−290				
950	2.48	335	1.58	−290				
1000	2.31	330	1.66	−290				

**Table 2:** Comparison of optimal *A*_*n*_/*A*_*p*_ ratios between 3D simulation and estimation from Equation (14).

Temperature range	300 K–500 K			300 K–800 K			300 K–1000 K
N-type	1	2	3	1	2	3	1
Optimal ratio from simulation	0.90	0.36	0.56	0.77	0.25	0.56	0.66
Optimal ratio from Eq. (14)	0.89	0.35	0.52	0.76	0.25	0.52	0.68
Difference	−1.1%	−2.8%	−7.1%	−1.3%	0.0%	−7.1%	3.0%
