# Supplementary material for: Modelling of segmented high-performance thermoelectric generators with effects of thermal radiation, electrical and thermal contact resistances
Source: Sci Rep. 2016 Apr 7;6:24123. doi: 10.1038/srep24123 (PMC4823787; doi:10.1038/srep24123)
Supplement: Supplementary Information [file srep24123-s1.pdf]

Supplementary Information

Modelling of segmented high-performance thermoelectric generators with effects of thermal radiation, electrical and thermal contact resistances

*Zhongliang Ouyang and Dawen Li**

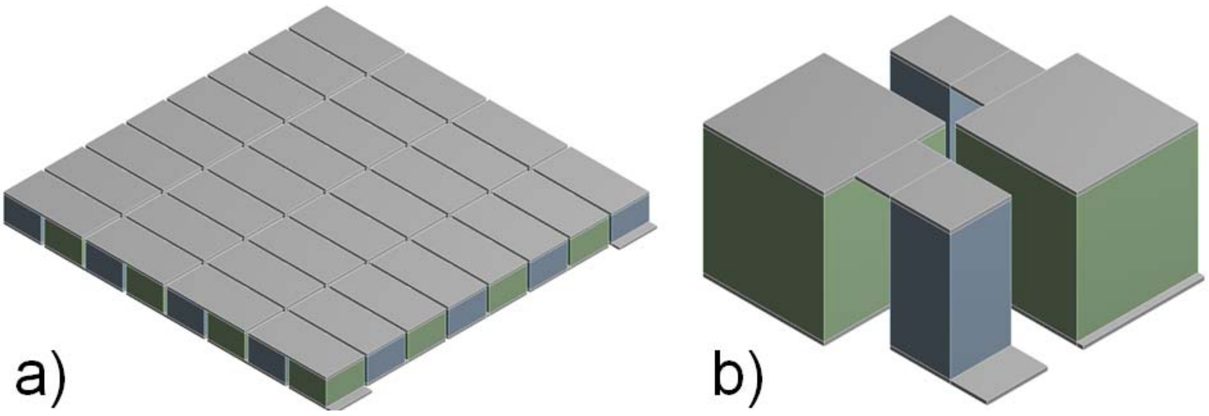

Figure S1 Illustration of a) symmetrical model with 8×8 legs, and b) non-symmetrical model with 2×2 legs used in simulation.

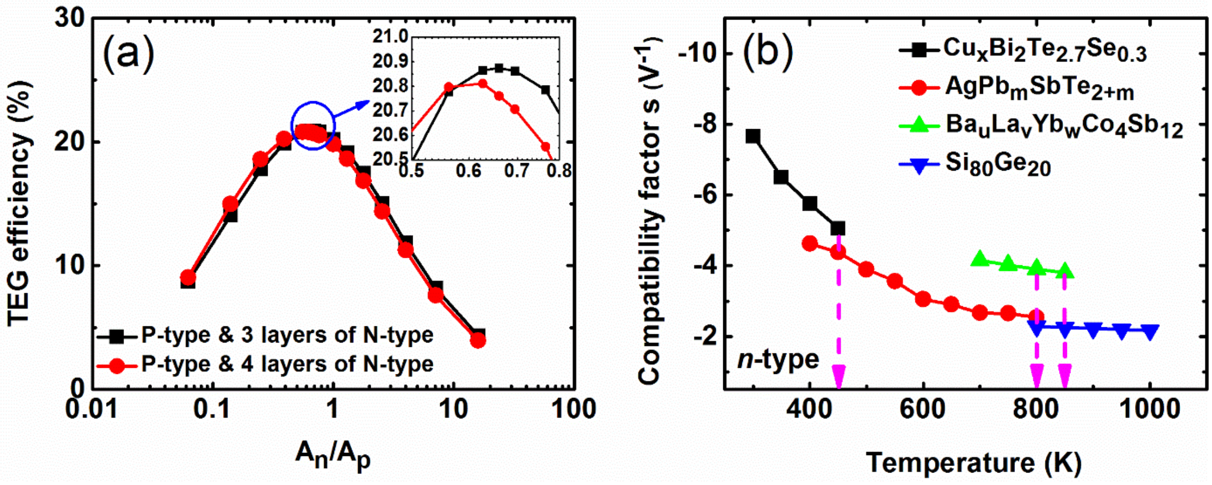

Figure S2 a) TEG Efficiency vs. geometrical ratios of the best p-type teaming with 3 or 4 layers of the strongest n-type TE materials @ ΔT = 700 K. b) Compatibility factors of the $BaLaYbCo_4Sb_{12}$ layer in comparison with the other three layers.

Table S1 Temperature dependence of resistivities ρ ($\times 10^{-5}$ Ω m) and thermal conductivities κ (W m^{-1} K^{-1}) for the used TE materials

	p-type		n-type 1		n-type 2		n-type 3	
	300-400K BiSbTe[1]<br>450-500K MgAgSb[2]<br>550-800K KPbTeS[3]<br>850-1000K SnSe[4]		300-400K CuBiTeSe[5]<br>450-800K AgPbSbTe[6]<br>850-1000K SiGe[7]		YbCoSb[8]		Pb(S,Se,Te)[9]	
T (K)	ρ	κ	ρ	κ	ρ	κ	ρ	κ
300	0.80	1.20	1.00	1.00	0.420	2.8	0.49	2.18
350	1.05	1.10	1.20	1.01	0.455	2.8	0.56	1.88
400	1.35	1.05	1.30	1.06	0.495	2.8	0.67	1.67
450	2.40	0.75	1.33	1.55	0.506	2.8	0.84	1.50
500	2.20	0.80	1.75	1.45	0.543	2.8	1.06	1.37
550	2.75	0.71	2.22	1.35	0.557	2.8	1.25	1.25
600	3.60	0.64	3.13	1.25	0.580	2.85	1.55	1.15
650	4.45	0.60	3.70	1.17	0.615	2.90	1.90	1.08
700	5.31	0.59	4.50	1.1	0.644	2.95	2.38	1.00
750	5.53	0.59	4.76	1.05	0.659	3.05	3.00	0.97
800	5.75	0.60	5.10	1.05	0.679	3.10	3.50	0.95
850	11.8	0.35	6.3	0.8				
900	11.8	0.35	6.3	0.8		N/A		
950	12.7	0.34	6.1	0.83				
1000	14.3	0.33	5.9	0.86				

Table S2 Temperature dependence of resistivity (Ω m) and thermal conductivity (W m^{-1} K^{-1}) for copper contacts

T (K)	Resistivity	Thermal conductivity
300	1.73E-08	397
500	3.02E-08	388
800	4.97E-08	370
1000	6.26E-08	357

Table S3 Dimensions of non-symmetrical models

Non-symmetrical models (L=10 mm, 2x2 legs)		
Group 1 ($A_p > A_n$) A_p=9.6×9.6mm^2 A_n(mm^2)	Group 2 ($A_p < A_n$) A_n=9.6×9.6mm^2 A_p(mm^2)	Total cross-section area (mm^2)
2.4×2.4	2.4×2.4	195.84
3.6×3.6	3.6×3.6	210.24
4.8×4.8	4.8×4.8	230.4
6.0×6.0	6.0×6.0	256.32
7.2×7.2	7.2×7.2	288
8.4×8.4	8.4×8.4	325.44
9.6×9.6	9.6×9.6	368.64

1 Xie, W. *et al.* Identifying the specific nanostructures responsible for the high thermoelectric performance of (Bi, Sb)$_2$Te$_3$ nanocomposites. *Nano Lett.* **10**, 3283-3289 (2010).

2 Zhao, H. *et al.* High thermoelectric performance of MgAgSb-based materials. *Nano Energ.* **7**, 97-103 (2014).

3 Wu, H. *et al.* Broad temperature plateau for thermoelectric figure of merit ZT>2 in phase-separated PbTe$_{0.7}$S$_{0.3}$. *Nature Comm.* **5**, 4515 (2014).

4 Zhao, L.-D. *et al.* Ultralow thermal conductivity and high thermoelectric figure of merit in SnSe crystals. *Nature* **508**, 373-377 (2014).

5 Liu, W. S. *et al.* Thermoelectric Property Studies on Cu-Doped n-type Cu$_x$Bi$_2$Te$_{2.7}$Se$_{0.3}$ Nanocomposites. *Adv. Energy Mater.* **1**, 577-587 (2011).

6 Hsu, K. F. *et al.* Cubic AgPbmSbTe$_{2+m}$: bulk thermoelectric materials with high figure of merit. *Science* **303**, 818-821 (2004).

7 Basu, R. *et al.* Improved thermoelectric performance of hot pressed nanostructured n-type SiGe bulk alloys. *J. Mater. Chem. A* **2**, 6922-6930 (2014).

8 Dahal, T. *et al.* Thermoelectric property enhancement in Yb-doped n-type skutterudites Yb$_x$Co$_4$Sb$_{12}$. *Acta Mater.* **75**, 316-321 (2014).

9 Aminorroaya Yamini, S. *et al.* Thermoelectric Performance of n-Type (PbTe)$_{0.75}$(PbS)$_{0.15}$(PbSe)$_{0.1}$ Composites. *ACS Appl. Mater. Interfaces* **6**, 11476-11483 (2014).
